# A High Rate of Non-Compliance Confounds the Study of Whole Grains and Weight Maintenance in a Randomised Intervention Trial—The Case for Greater Use of Dietary Biomarkers in Nutrition Intervention Studies

**DOI:** 10.3390/nu9010055

**Published:** 2017-01-11

**Authors:** Mette Kristensen, Xavier Pelletier, Alastair B. Ross, Frank Thielecke

**Affiliations:** 1 Department of Nutrition, Exercise and Sports, University of Copenhagen, Copenhagen DK2200, Denmark; mekr@life.ku.dk; 2 Optimed Clinical Research, Gieres 38610, France; xavier.pelletier@optimed.fr; 3 Nestlé Research Center, Vers chez les Blanc, Lausanne 1800, Switzerland; 4 Department of Biology and Biological Engineering, Chalmers University of Technology, Gothenburg 41296, Sweden; 5 Cereal Partners Worldwide, Orbe 1350, Switzerland; frank.b.thielecke@gmail.com; 6 T2 Goodness Ltd., Allschwil 4123, Switzerland

**Keywords:** whole grain, weight maintenance, compliance, biomarkers, alkylresorcinols

## Abstract

Observational studies consistently find an inverse relationship between whole-grain intake and weight gain. We aimed to confirm this in an open-label researcher-blinded parallel design randomised trial. A total of 179 overweight/obese women with a habitually low whole-grain intake (<16 g/day) were randomised to a weight maintenance diet with refined-grain (RG) or whole-grain (WG) foods (80 g/day) for 12 weeks after an initial weight loss program over 8 weeks. Body weight and composition was assessed at baseline, after the initial weight loss, and after the 12-week dietary intervention. During the 12-week dietary intervention phase, there were no group differences in changes in body weight and total fat mass %, whereas abdominal fat mass tended to increase more during the dietary intervention phase in the WG compared to the RG group (0.7 (SD 3.6) vs. −0.3 (SD 3.8) %; *p* = 0.052). Plasma alkylresorcinol concentrations, biomarkers of wholegrain wheat and rye intake, indicated poor compliance, particularly in the WG group, where >60% of participants had alkylresorcinol concentrations below 70 nmol/L, a concentration indicating low or no intake of whole-grain wheat. Further, weight regain was lower than expected in both intervention groups, further supporting a lack of compliance to the post-weight-loss diet. The rate of compliance was too low to conclude any effect of whole grain on weight maintenance, and reinforces the need to use objective measures of compliance in nutrition intervention studies.

## 1. Introduction

Greater whole-grain intake is associated with reduced weight gain and lower body mass index (BMI) in several observational studies [1,2,3,4,5,6,7,8,9,10,11,12,13,14,15,16,17], though not all [18,19,20,21]. Other observational studies have found a healthier body fat distribution is also associated with greater intake of whole grains [15,22]. While the effect size is small (around 0.3 kg less weight gain for highest vs. lowest intakes of whole grains [6]), this may still have an important impact on the prevention of obesity related diseases. Although the inverse relationship between intake of whole grains and weight gain is generally consistent, there is still room for residual confounding due to the association of whole-grain intake with a healthy lifestyle, even if this has been factored into the models. These associations have been tested in several intervention trials of varying designs to determine if whole grains may aid weight loss or loss of fat. Results from these trials are mixed, with some studies indicating greater weight loss or loss of body fat when subjects are fed whole grains rather than refined grains [23,24,25,26], and others finding no advantage [27]. A recent meta-analysis of intervention trials examining whole grains and their effect on weight loss and body composition found that there was no overall effect on weight, but a small effect for increasing fat loss (−0.48%) in the limited number of trials that have assessed this variable [28].

Study designs vary widely among these studies, which have included whole grain intake against a background of both hypocaloric and isocaloric diets, different amounts of whole grains, and different types of grain. This heterogeneity in design complicates the overall evaluation of the evidence, and also raises an additional problem in that study design and location/population potentially have a major effect on dietary compliance. Katcher et al. identified the problem of subjects adding whole grains to their diet, rather than replacing current foods with whole grains [24], an issue that was also highlighted by Brownlee et al. [29] in a large-scale intervention of nearly 300 subjects. This leads to increased overall energy intake, which would nullify any benefit of choosing whole grains over refined grains. The issue of compliance in nutritional intervention studies is well known, especially in free-living settings, and over longer periods of time [30,31,32,33]. Using biomarkers of food intake has been proposed as one way of ensuring compliance in whole-grain intervention studies [34], and by measuring plasma alkylresorcinols, it is possible to monitor likely compliance to whole-grain interventions based on wheat and rye. Ideally, a biomarker should be used together with a validated dietary assessment tool, though there are several issues with this approach—mainly what compliance measurement tool should take precedence: self-reported recall or a non-subjective measurement from a biofluid, and how to handle wide variation in intra-individual biomarker response. To date, plasma alkylresorcinols have been used as biomarkers of compliance in several studies [23,35,36,37]. In these four studies, diet effects were observed for primary or secondary outcomes, and compliance as determined by plasma alkylresorcinol concentrations was high. While relatively expensive to use for large-scale intervention trials, it is possible that plasma alkylresorcinols can provide an added level of surety when assessing the results of nutrition intervention trials.

In order to assess whether findings from observational studies that whole grains reduce weight gain can be replicated in an intervention trial setting, we performed a randomised parallel design researcher-blinded intervention trial in overweight and obese women where the primary objective was to assess abdominal fat regain over three months after an initial weight loss. Secondary outcomes were changes to body weight, clinical chemistry markers, and plasma alkylresorcinols as well as nutritional intake and gastrointestinal symptoms.

## 2. Methods

### 2.1. Study Design

**Figure 1:**
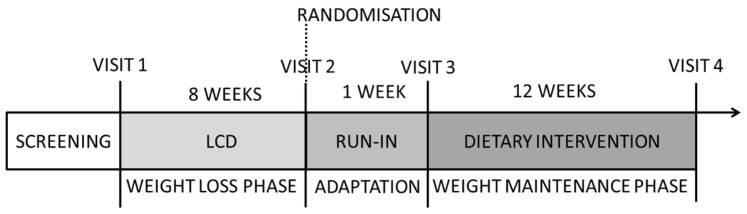
Experimental design of the study. LCD: low-calorie diet.

The study was a parallel group design based at a single study centre (Grenoble, France). The study was divided into three phases. The study design is presented graphically in Figure 1. During the first phase, participants were instructed to follow a low-calorie diet (LCD) (1200 kcal/day) with the aim to lose 6% of their body weight in 8 weeks. Participants who were successful in this goal (*n* = 178) were then randomised onto one of two ad libitum diets by block-randomisation (block size, *n* = 4). There was a 1-week run-in period between the LCD phase and the start of the dietary intervention, where all participants were provided with refined-grain foods (and no whole-grain foods) to put them on an equal starting point for cereal food intake after the weight loss phase. The two intervention diets were based on either eating at least 80 g of whole grain per day or the equivalent as refined grains for 12 weeks, based on foods provided by the study centre. The participants and dieticians who assessed compliance to the intervention diets were not blinded during the intervention, but outcome assessors as well as statisticians were blinded until data analyses were completed. The study was approved by both the regional ethical committee (C.P.P. Sud-Est III) and the Health Authorities (ANSM), and carried out according to the Declaration of Helsinki. All subjects provided written informed consent for participation in the study. The study was registered at clinicaltrials.gov (NCT01239147).

### 2.2. Participants

Overweight and obese (body mass index (BMI): 27–34 kg/m^2^) women between the ages of 20 and 50 were recruited for this study from the Grenoble region of France. Only women were selected for this trial in order to reduce potential heterogeneity due to gender. Participant inclusion criteria were age (20–50 years), healthy (determined by clinical examination and medical history), pre-menopause, BMI (27–34 kg/m^2^), waist circumference (>80 cm), no special diet in the previous 3 months, normal blood pressure and heart rate (systolic 95–140 mmHg; diastolic 50–90 mmHg), low habitual whole-grain intake (<16 g/day determined using a previously validated questionnaire for a French-speaking population [38]). Exclusion criteria beyond being out of the range of the inclusion criteria were: history of metabolic disease including type 2 diabetes, renal insufficiency, taking medication or supplements to aid weight loss, a variable medication regime, pregnancy or breast feeding, participation in a remunerated clinical trial within the previous 12 months, and known food allergies of relevance to the project.

### 2.3. Experimental Diet

During the weight loss phase, participants were provided with commercially available weight loss products (Nestlé Protéika^®^, Nestlé SA, Paris, France), consisting of bars, shakes, and ready-to-eat meals, along with advice from a dietician on how to incorporate these foods into their diet and restrict their daily energy intake to 1200 kcal/day. The weight loss regime was the same for all participants, independent of body size and physical activity level. 

Following the LCD period, participants were randomised onto a whole-grain (WG) diet or a refined-grain (RG) diet, with dietary advice to help them to maintain their weight. Whole-grain and refined-grain products were provided to the subjects in a supermarket setting (“shop”) at the study site, where they could take products ad libitum. The shop was divided into two sections: one with whole-grain products, the other with refined-grain products. Subjects came to the study site once per week to choose food items appropriate for their dietary group, and a dietician recorded all food items taken from the shop. Whole grain was defined based on the Health Grain Forum definition [39], that all parts of the grain were present in the products in their correct proportions, allowing for some minor losses of bran. The proportion of whole grains in products was based on that reported by manufacturers on the product packaging, and confirmed by analysis of alkylresorcinols in the case of the wheat and rye products [40]. Participants in the WG group were asked to include at least 80 g of whole grains per day during the WG period. The whole-grain products provided included bread, breakfast cereal, pasta, rice, couscous, and muesli bars; each product had between 25% and 100% whole grain by weight. Participants on the refined-grain diet were provided equivalent non-whole-grain foods to select from. Details of the foods provided in the study are in Supplementary Materials Table S1.

### 2.4. Nutritional Counselling

During all phases of the study, the participants met with a trained dietician to receive nutritional advice on a weekly basis. In case of holiday or similar events, the dietary counselling was done by telephone. During the 8-week LCD period, they were instructed on how to keep to a calorie-restricted diet (1200 kcal/day) and incorporate the LCD products into their habitual diet. During the 12-week weight-maintenance period, participants in both treatment groups received nutritional counselling on how to incorporate the refined-grain and whole-grain products, respectively, into their diet and not to change their dietary habits beyond this. Particular attention was paid to helping subjects on the whole-grain intervention of replacing refined-grain food items in their diet by emphasising substitution rather than addition of these foods. Furthermore, all participants were instructed to maintain their physical activity level throughout the study. Individual energy requirements were assessed using the Schofield equation based on weight reached after the weight loss phase and assuming a low activity factor (1.3). The same level of dietary counselling was offered to all participants, irrespective of group allocation.

### 2.5. Dietary Intake Assessment

Intake of refined-grain and whole-grain foods was monitored by review of daily recordings done by the participants. Furthermore, participants filled in a 3-day food record prior to the start of the study (visit 1) and towards the end of the dietary intervention (visit 4). Subjects also reported satisfaction with the diets using a validated 45-item questionnaire previously used in a whole-grain intervention study [24], and level of physical activity was assessed using Baecke’s short questionnaire [41] at visits 1, 3 and 4.

### 2.6. Anthropometric Measurements

All measurements were performed in the morning after ≥10 h fasting and after voiding of the bladder. Body weight was measured on an electronic scale to the nearest 0.1 kg (TBF300, TANITA Corp., Tokyo, Japan), while the participants were wearing light clothing and no shoes. Height was measured to the nearest 0.5 cm by using a wall-mounted stadiometer without shoes. Waist circumference was measured midway between the anterior superior iliac spine and the base of the last rib, while hip circumference was measured at the widest posterior extension of the buttocks. Both were measured to the nearest 0.5 cm. Sagittal abdominal diameter (SAD) was measured once to the nearest 0.1 cm. Body composition was measured before and after the dietary intervention period by dual-energy X-ray absorptiometry (DEXA) scanning (Lunar Radiation Co., General Electric, Madison, WI, USA), and fat-free mass (FFM) was calculated as total body mass − fat mass (FM). Abdominal fat mas was directly computed using anatomical reference measures, and reported as % of total fat as measured by DEXA.

### 2.7. Biochemical Measurements

Blood samples were collected with both lithium heparin and ethylenediaminetetraacetic acid (EDTA) as anticoagulants, and plasma was separated by centrifugation at 1000× *g* for 10 min at 4 °C. Samples were aliquoted and stored at −80 °C before analysis. Plasma total cholesterol, high-density lipoprotein (HDL) cholesterol, triglycerides, glucose, high-sensitivity C-reactive protein (hsCRP), glycated haemoglobin, insulin, leptin, adiponectin, and plasminogen activation inhibitor 1 (PAI-1) were measured using validated standard enzyme linked immunoassays. All clinical chemistry parameters were analysed in a blinded manner by a third-party laboratory. Plasma alkylresorcinols were measured using normal-phase liquid chromatography coupled to a tandem mass spectrometer using a previously validated method [42]. Plasma total alkylresorcinol concentrations >70 nmol/L were taken as a conservative cutoff for compliance for both groups, based on data from controlled diet studies [34]. Alkylresorcinols in study foods were analysed using high-performance liquid chromatography with fluorescence detection [40].

### 2.8. Statistical Analyses

Sample size estimation was based on demonstrating a difference in mean body fat mass maintenance after an initial loss of fat mass at a significance level of 5% and with a statistical power of 80%. The initial calculations suggested that by including 80 participants in each group, we would be able to detect a difference in body fat mass of 1.8 (SD 4.0) kg. In addition, we anticipated a dropout rate of <10% during the dietary intervention, thus we aimed to enrol at least 88 participants into each group. Due to a lack of previous studies on whole grains and abdominal fat loss at the time the study was planned, power calculations were only done for loss of total body fat. All statistical analyses were performed in SAS^®^ System for Windows (release 9.4, SAS Institute Inc., Cary, NC, USA). Homogeneity of variance and normal distribution were inspected using residuals plots, normal probability plots, and histogram.

Group comparisons of variables assessed at baseline (visit 1) and prior to the dietary intervention (visit 3) as well as dropout rate were done using a one-way ANOVA or Mann–Whitney Wilcoxon tests, as appropriate, for quantitative parameters, and a chi-squared (χ^2^) or Fisher’s exact test, as appropriate, for qualitative parameters. Group differences in changes in outcomes assessed during the LCD phase (between visits 1 and 2) and during the dietary intervention (between visits 3 and 4) were assessed using a one-way ANCOVA with group (RG or WG) as a fixed variable and corresponding baseline (visit 1 or 3) value and age as a covariate. Both complete case (CC) and intention-to-treat (ITT) analyses were performed, but CC analyses were considered the primary analyses of interest. All data are presented as means with SD unless otherwise indicated. Differences were considered significant at *p* < 0.05. 

## 3. Results

### 3.1. Flow of Participants during the Study

In total, 317 overweight or obese women were enrolled into the weight loss phase, of which 178 obtained a weight loss of ≥6% and were eligible for randomisation (Supplementary Materials Figure S1). Thus, a total of 178 women were randomised to the RG group (*n* = 89) or the WG group (*n* = 89), respectively. The dropout rate during the dietary intervention differed between the two groups (*p* = 0.034), as 1 and 8 participants dropped out from the RG and WG groups, respectively. Even so, there was no difference between ITT analyses and CC analyses in any of the outcomes assessed, thus only results from the CC analyses are reported here. Reasons for subject dropouts were 6 premature withdrawals, 2 subjects with positive pregnancy tests, and 1 inability to comply with the study diet. Baseline characteristics of the subjects are presented in Table 1.

### 3.2. Dietary Intake and Compliance

**Figure 2:**
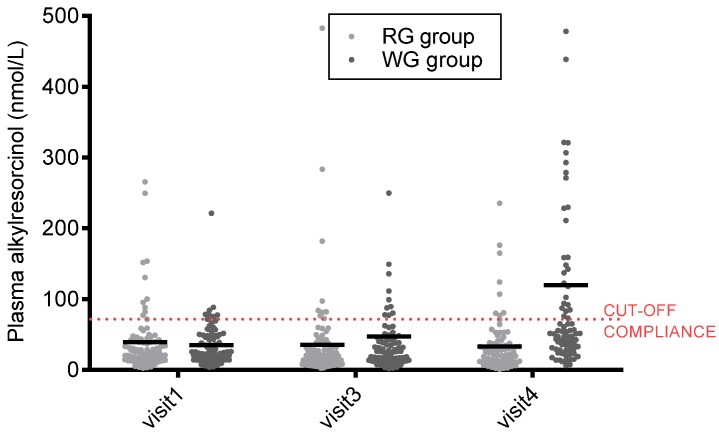
Mean and individual plasma alkylresorcinol concentration (nmol/L) for individuals by intervention at visit 1 (at baseline), visit 3 (after the low-calorie diet (LCD) and run-in periods), and visit 4 after the 12-week dietary intervention with either refined (RG) or wholegrain (WG) foods. For clarity, four subjects with plasma alkylresorcinol concentrations >500 nmol/L at visit 4 in the WG group have not been included.

**Figure 3:**
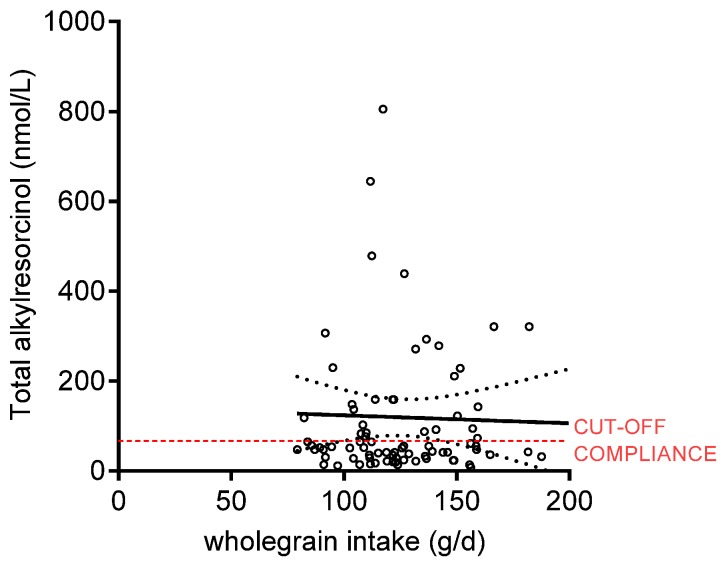
Correlation between reported mean whole-grain intake (g/day) during the 12-week dietary intervention and plasma alkylresorcinol concentration (nmol/L) measured at visit 4 with 95% confidence intervals for participants in the WG group (*n* 81).

Dietary intake data based on 3-day food records are presented in Table 2. There was no difference at baseline for total energy and macronutrient intake. During the intervention, there was a higher reported intake of dietary fibre and lower reported intake of fat in the WG group. Mean daily intake of whole-grain foods in the RG and WG group after 12 weeks was reported to be 0.5 (SD 1.6) g and 124 (SD 1.7) g, respectively (*p* < 0.0001). Based on a detailed diet analysis on a random selection of six participants, the average (min; max) proportion of grains eaten on the WG diet was 79 (64; 90) % wheat, 16 (6; 29) % rice, 4 (0; 12) % oats, and 1 (0; 6) % rye. The main grains eaten on the RG diet were wheat and rice. Plasma alkylresorcinol concentrations were 119 (SD 181) nmol/L in the WG group and 33.6 (SD 38.9) nmol/L in the RG group after the dietary intervention, and the change from visit 3 was significantly different between groups (*p* < 0.0001) (Table 3), even though 62% of the participants in the WG group had plasma alkylresorcinol concentrations <70 nmol/L, which was applied as a conservative cutoff for compliance (Figure 2). Plasma alkylresorcinols and reported whole-grain intake in the WG group after the dietary intervention (visit 4), however, did not correlate (Spearman’s correlation coefficient *R* = −0.02919; *p* = 0.85), which indicates poor compliance (Figure 3).

There was no difference in the overall diet satisfaction score prior to the dietary intervention phase (visit 3) (3.7 (SD 0.5) vs. 3.7 (SD 0.4) in the RG and WG groups, respectively; *p* > 0.80). Both diets were equally well accepted, as a small, but significant increase was seen in both groups during the intervention, but no group difference was present (0.1 (SD 0.3) vs. 0.1 (SD 0.4) in the RG and WG groups, respectively; *p* > 0.80).

### 3.3. Anthropometric Measures

Initial body weight and total and abdominal body fat % did not differ between groups (*p* = 0.27, *p* = 0.30, and *p* = 0.16), and during the LCD phase the women lost 7.8 (SD 2.0) % and 7.6 (SD 1.9) % of their body weight and 3.3 (SD 2.4) % and 3.8 (SD 2.2) % total body fat mass in the RG and WG groups, respectively (Table 4). A slightly larger decrease, albeit not significant, in abdominal fat % was seen during the LCD among women randomised to the WG group compared to those randomised to the RG diet (−5.0 (SD 3.6) % vs. −4.2 (SD 3.7) %; *p* = 0.09), whereas changes in loss of body weight (*p* = 0.48) and total fat mass % (*p* = 0.11) during the LCD phase did not differ between groups. 

**Figure 4:**
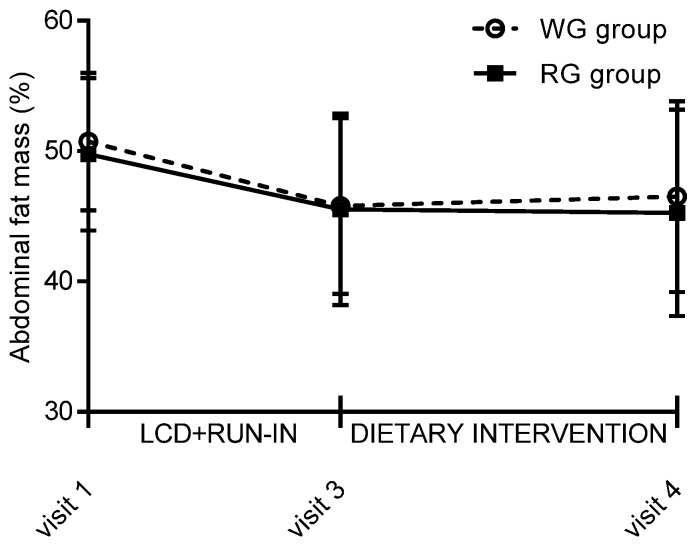
Mean abdominal fat mass percentage with SD during the LCD and run-in phases (visit 1–3) and dietary intervention phase (visit 3–4). WG, whole grain; RG, refined grain; LCD, low calorie diet.

During the dietary intervention phase, body weight increased slightly in both groups, but changes did not differ between groups (0.36 (SD 2.7) kg and 0.43 (SD 2.3) kg in the RG and WG groups, respectively; *p* = 0.96. Further adjustment for body weight loss during the LCD phase did not change this result. In contrast, abdominal fat mass tended to increase more during the dietary intervention phase among women randomised to the WG compared to the RG group (0.7 (SD 3.6) % vs. −0.3 (SD 3.8) %; *p* = 0.052) (Figure 4). Further adjustment for loss of abdominal fat mass during the LCD phase only slightly attenuated this finding (*p* = 0.062). However, changes in total fat mass % during the dietary intervention did not differ between groups (*p* = 0.11), and adjustment for loss of fat mass during the LCD phase did not change the result (*p* = 0.11). Finally, changes in fat free mass, BMI, waist and hip circumference, or SAD did not differ between the RG and WG groups.

Post hoc, we explored the indication of poor compliance in the WG group by performing regression models in order to identify the main determinants of loss of total and abdominal body fat. Independent of diet allocation, plasma alkylresorcinols (at visit 4) were not associated with loss of total or abdominal fat mass, nor body weight (data not shown). Furthermore, due to the potential lack of compliance according to plasma concentration concentrations of alkylresorcinols, an analysis on only the subgroup of compliant participants was conducted (<70 nmol/L for RG and >70 nmol/L for WG at visit 4). For change of abdominal fat mass % during the dietary intervention, the difference between groups was further attenuated when only including compliant participants (RG: *n* 76; WG: *n* 30) in both groups (−0.44 (3.81) vs. 0.45 (3.95) % in the RG vs. WG group, respectively; *p* = 0.095), and not present among non-compliant participants. For change in body weight, a stratified analysis did not change the result (0.37 (2.77) vs. 0.65 (2.44) % in the RG vs. WG group, respectively).

### 3.4. Cardiometabolic Outcomes

After 12 weeks of dietary intervention, there was no difference between the RG and WG groups in changes of blood pressure, blood lipids, inflammatory markers, or markers of glucose metabolism (all *p*-values > 0.20) (Table 3). We note here that the menstrual cycle was not controlled for, which can have a major impact on the variability of these outcomes [43], and may obscure any findings beyond those highlighted regarding compliance.

## 4. Discussion

In a study designed to investigate whether weight regain after weight loss could be reduced by eating whole grains instead of refined grains, we found that >60% of the subjects randomised to the whole-grain diet were unlikely to have complied to the diet. This prevents any conclusions on the relationship between whole grains and the primary and secondary endpoints measured, but does highlight the importance of using biomarkers of intake to check compliance in nutrition intervention studies. Had these not been used, the only conclusion possible, that whole grains were found not to have any effect on weight regain, would in fact not be supported by our trial based the numbers who actually followed the prescribed protocol.

The use of biomarkers to monitor compliance in intervention studies can be controversial, especially if other measures of compliance (e.g., diaries or questionnaires) indicate excellent compliance, as was the case in this study. Which measure takes precedence? As plasma alkylresorcinols are non-subjective, they are not affected by a subject’s perception of what they should be doing, whereas diaries or questionnaires would be expected to be heavily influenced by preconceived ideas of what is required when reporting to the study personnel. In this case, plasma alkylresorcinols may better reflect what is actually going on and be a more reliable indicator of compliance. Several studies have established that plasma alkylresorcinols do respond rapidly to intake of wholegrain wheat and rye [35], and that regular consumption of whole grains under controlled conditions leads to stable and repeatable elevated fasting plasma concentrations [44]. While inter-individual variation is high, and many factors can influence plasma alkylresorcinol concentration beyond intake of whole grains [35,45], it is highly unlikely that someone eating 80 g of whole-grain foods, mainly wheat, would have plasma total alkylresorcinol concentrations <70 nmol/L. Plasma alkylresorcinols would not detect if a person ate mainly oat- or rice-based products, though subjects on the whole-grain intervention reported eating mainly wheat products, the minimum amount being 64%, equivalent to 50 g/day of whole-grain wheat. Even if a subject only chose to eat all their whole grain at breakfast time, giving the maximum amount of time for concentrations to return to baseline, fasting concentrations still remain higher 24 h later than a non-whole-grain diet (Ross et al., under preparation). Although on a group basis there was a difference in plasma alkylresorcinol measurements, this was largely driven by relatively few subjects with very high concentrations. A similar result was seen in another large study where whole-grain foods were provided in an open setting, where although mean alkylresorcinol concentrations differed, a large proportion of individuals had very low concentrations that would be unlikely based on results from controlled whole-grain trials where alkylresorcinols have been measured [35]. So, while it is not possible to rule out that subjects were actually compliant even with low plasma alkylresorcinol concentrations, based on the high proportion of whole-grain wheat products consumed in the detailed analysis of a subset of subjects, the overall high amount of whole grains reported as being eaten (mean 124 g/day) and what is known from controlled whole-grain feeding studies, we have to raise strong doubts about the subjects’ compliance to the diet. The alkylresorcinol concentrations suggest that compliance was so low that no conclusions on the relationship of whole grains and weight regain after weight loss that can be made from this study.

If there was an apparent problem of compliance to the whole-grain diet, it is necessary to attempt to explain why. Personalised nutritional advice was provided to the subjects to maintain a healthy diet, and this may have aided subjects in not overeating after the weight loss period. This added awareness and support for eating a healthy diet could reduce any potential effect of whole grains on satiety or satiation compared to refined grains. Foods provided were commercially available in the country where the study was carried out, and considered to be organoleptically acceptable. No difference in taste preference or gastrointestinal comfort between the diets was reported by the participants, although the higher dropout rate in the WG group may indicate that the subjects were less willing to eat foods that were not part of their habitual diet. Another factor may be that subjects, having lost weight in the first part of the study, attempted to maintain a reduced energy diet to maintain the weight loss, and avoided eating the study products they collected. This is supported by the lack of any appreciable weight gain over the 3-month diet intervention period. The energy derived from the intervention products would have made up a considerable proportion of their total intake when compliant, and not consuming the intervention products, or consuming them in reduced amounts, would have a major effect on overall energy intake. It has previously been shown that a high intake of intervention foods providing ~100 g whole grains per day is possible during weight loss [23]. The design applied here was different, however, as an 8-week LCD phase was applied prior to the dietary intervention, where differences in weight regain were targeted. A low weight regain during the 12-week intervention period was seen in both groups, which makes it difficult to detect potentially subtle differences between groups. A similar design was applied in the DIOGENES pan-European study, but weight regains were larger, and the intervention period lasted 6 months rather than 12 weeks [46]. Thus, a longer study period may have produced different results. Finally, the French population are not habitual whole-grain consumers in general [47] and the participants were recruited on the basis of being non-whole-grain consumers (<16 g whole grains/day), as we expected a change towards a high whole-grain intake to benefit non-consumers the most. The fact that the participants were not accustomed to the whole-grain foods may have been a disadvantage rather than an advantage, as it may affect the participants’ willingness to incorporate the unfamiliar foods into their diet, resulting in poor compliance. Supporting this theory, a study carried out by Kristensen et al. (2012) in Denmark [23], where the whole-grain intake is among the highest in the world, did not prevent significant differences being observed resulting from participants eating a whole-grain or refined-grain diet. In the U.K., the WHOLEHEART study was conducted in a group of ~300 non-whole-grain consumers [29]. Here, the participants were allocated to three different groups (0, 80, and 160 g of whole grains/day), and they found that particularly the participants consuming the most whole grain tended to increase their overall energy intake, indicating that substitution of habitual foods was difficult. In the present study, the dietary intake data do not, however, show that participants in the WG group consumed more energy than those randomised to the RG group. Given that the same dietary data indicated that average whole-grain intake was >100 g/day, we have reason to doubt the reliability of the dietary intake data. Beyond an issue with compliance to the whole-grain diet, is that neither group gained the expected amount of weight after the 3-month dietary intervention. This suggests that subjects in both groups were not compliant, adding weight to the hypothesis that lack of compliance was due to a desire of the subjects to maintain weight loss, rather than a specific aversion to the whole-grain products provided. Although plasma alkylresorcinols can detect the difference between whole-grain, refined-grain wheat, and gluten-free diets [48], they would be unlikely to detect non-compliance to the control diet, as all subjects were likely to be regular consumers of refined wheat-based foods since they were recruited on the basis of being low-whole-grain consumers. Further, there was no difference between plasma alkylresorcinol measurements at any of the three time points for the control group.

Although compliance was highlighted as a problem in this study, analysis using ITT or CC did not change any of the outcomes. These do not reflect the measured compliance according to plasma alkylresorcinols, so post hoc analyses were performed, using plasma alkylresorcinol concentrations in place of whole-grain intake based on diet records. As the number of subjects that could be considered to be compliant was low (*n* = 106), the resulting statistical power of the post hoc analyses was far lower than that calculated as necessary to find a difference between groups. Weak trends were observed for regression models for plasma alkylresorcinols and a reduction in body fat, but these were not consistent between different measures of abdominal fat, suggesting that the available power from compliant subjects was insufficient for the study outcome.

This study highlights the broader issue of inconsistency of results from dietary intervention studies—both for whole grains [49] and in general. While most studies do include compliance checks—including diaries, questionnaires, and collection of empty packaging—unless the subjects are observed eating their intervention products, it is very difficult to guarantee if subjects have actually followed the requested protocol, including whether they have replaced or added intervention foods to their regular diet. Although dietary biomarkers are frequently called for, their actual use in intervention studies remains limited, in part because although there is much work on discovery of these biomarkers, there is less work on their validation—research which is essential for biomarkers to be used to ensure compliance. While biomarkers are useful for ensuring that study outcomes are trustworthy from a compliance perspective, it should be remembered that they remain an “ambulance at the bottom of the cliff”—once they highlight the problem, the study is likely to have been finished long ago. More work needs to be done during a study to ensure compliance, and under ideal circumstances, biomarker measurements should be made during the course of a long-term study so that direct feedback can be given to the subjects. Although there are many barriers to this at present, with time it could become standard procedure to check compliance based on several biomarkers throughout the course of a nutrition intervention study.

## 5. Conclusions

In conclusion, this study, designed to test if a whole-grain diet leads to lower body weight regain after weight loss, was hampered by a high level of non-compliance, and does not allow any conclusion to be made about the role of whole grains and body weight and body composition. This study does highlight the importance of including biomarkers of intake in nutrition intervention studies, which has provided clear evidence that the study has not tested the hypothesis it was designed to do. We are able to highlight a weakness in this type of study design if subjects are highly motivated to maintain weight loss, and the consequent importance of incorporating a biomarker of food intake to allow post hoc validation of compliance. Further studies are needed to address the tantalising findings of some intervention studies that whole grains may positively impact on body composition, and these should place extra weight on ensuring compliance to the recommended diet beyond self-report during the study.

## Figures and Tables

**Table 1:** Baseline characteristics of the participants (visit 1) in the whole-grain (WG) and refined-grain (RG) groups (mean values and standard deviations (SD)).

	RG Group (*n* = 88)		WG Group (*n* = 81)	
	Mean	SD	Mean	SD
Age (years)	35.3	8.7	36.2	10.1
Height (cm)	164.6	6.7	163.0	6.1
Weight (kg)	81.5	8.1	80.2	7.2
Body mass index (kg/m^2^)	30.1	2.0	30.2	1.9
Total body fat (%)	44.5	4.8	45.5	4.4
Abdominal body fat (%)	45.6	7.4	45.6	6.7
Whole-grain intake (g/day)	13.5	29.9	13.0	25.3
Current smokers (*n*)	28		20	
Caucasian/Black/Asian/other (*n*/*n*/*n*/*n*)	81/5/1/1		76/4/0/1	

**Table 2:** Dietary intake data from 3-day food records of the participants at baseline (visit 1) and during the last week of the dietary intervention (visit 4) in the whole-grain (WG) and refined-grain (RG) groups (mean values and standard deviations (SD)).

	Baseline				During Dietary Intervention			
	RG Group (*n* 88)		WG Group (*n* 81)		RG Group (*n* 88)		WG Group (*n* 81)	
	Mean	SD	Mean	SD	Mean	SD	Mean	SD
Energy intake (kcal/day)	1759	472	1823	541	1402	396	1331	321
Protein (E%)	18.0	3.5	17.2	3.7	17.4	3.3	18.2	3.5
Fat (E%)	37.2	5.9	38.7	7.3	31.9	7.5	28.9 *	5.7
Carbohydrates (E%)	43.2	6.6	42.6	7.0	50.0	7.3	51.9	8.4
Dietary fibre (g/day)	13.6	5.2	13.8	5.1	13.3	4.1	18.5 *	5.2
Whole grain intake (g/day)	13.5	29.9	13.0	25.3	0.5	2.6	124 *	29

* Indicate significantly different changes in WG compared to RG group; *p* < 0.05.

**Table 3:** Mean values prior to (visit 3) and unadjusted changes in cardiometabolic outcomes and plasma alkylresorcinols during the dietary intervention phase (visit 4–visit 3) in the whole-grain (WG) and refined-grain (RG) groups (mean values with standard deviations (SD)).

	RG Group (*n* 88)				WG Group (*n* 81)			
	At Visit 3		Δ Visit 4–Visit 3		At Visit 3		Δ Visit 4–Visit 3	
	Mean	SD	Mean	SD	Mean	SD	Mean	SD
Systolic blood pressure (mmHg)	111.2	10.0	0.2	10.0	109.8	10.2	1.0	9.3
Diastolic blood pressure (mmHg)	71.1	7.4	−1.2	7.6	71.0	8.3	0.0	8.5
Glucose (mmol/L)	4.78	0.41	−0.05	0.35	4.79	0.39	−0.07	0.49
Insulin (pmol/L)	61.1	36.8	−1.8	32.0	55.9	29.5	1.8	28.4
Glycated haemoglobin, HbA1C (%)	5.34	0.29	0.02	0.20	5.29	0.31	0.04	0.17
Total cholesterol (mmol/L)	4.39	0.79	0.47	0.57	4.62	0.88	0.42	0.74
LDL cholesterol (mmol/L)	2.72	0.69	0.21	0.46	2.90	0.79	0.19	0.60
HDL cholesterol (mmol/L)	1.29	0.29	0.22	0.21	1.29	0.26	0.18	0.23
Total triacylglycerol (mmol/L)	0.86	0.36	0.08	0.37	0.95	0.45	0.09	0.40
Leptin (µg/L)	19.8	9.4	4.3	7.7	18.3	11.2	6.7	7.9
Adiponectin (mg/L)	9.32	4.54	0.97	2.42	8.39	6.32	1.30	2.16
High sensitivity C-reactive protein(mg/L)	3.00	3.88	−0.06	2.92	3.32	6.63	0.67	7.54
Plasminogen activator inhibtior-1 (mmol/L)	12.81	5.40	2.96	3.13	12.62	6.13	3.36	10.44
Alkylresorcinols (nmol/L)	35.6	62.9	−2.0	70.4	47.6	109.2	71.2	199.0 *

* Indicates significantly different changes in WG compared to RG group; *p* < 0.0001.

**Table 4:** Mean values prior to (visit 3) and unadjusted changes in anthropometric outcomes during the dietary intervention phase (visit 4–visit 3) in the whole-grain (WG) and refined grain (RG) groups (mean values with standard deviations (SD)).

	Refined Grain (*n* 88)				Whole Grain (*n* 81)			
	At Visit 3		Visit 4–Visit 3		At Visit 3		Visit 4–Visit 3	
	Mean	SD	Mean	SD	Mean	SD	Mean	SD
Body weight (kg)	74.9	7.7	0.4	2.7	73.9	6.6	0.4	2.3
Body Mass Index (kg/m^2^)	27.6	2.0	0.14	1.0	27.8	1.9	1.8	0.9
Total fat mass (%)	41.1	5.5	−0.2	2.9	41.7	5.0	0.4	2.3
Abdominal body fat (%)	45.6	7.4	−0.3	3.8	45.8	6.8	0.7	3.6
Sagittal abdominal diameter (cm)	19.5	1.7	0.1	1.2	19.3	1.7	0.3	0.9
Waist circumference (cm)	85.4	6.4	−0.1	4.2	85.6	6.8	0.0	3.2
Hip circumference (cm)	107.3	6.4	0.1	2.9	107.3	5.0	0.2	2.5

## Supplementary Materials

The following are available online at http://www.mdpi.com/2072-6643/9/1/55/s1. Table S1. Overview of intervention foods including whole-grain content and alkylresorcinol content used in the study in the wholegrain and refined grain groups; WG: whole grain; AR: alkylresorcinol; DM: dry matter. Figure S1. Flow of participants through the study.
