# Supplementary material for: A High Rate of Non-Compliance Confounds the Study of Whole Grains and Weight Maintenance in a Randomised Intervention Trial—The Case for Greater Use of Dietary Biomarkers in Nutrition Intervention Studies
Source: Nutrients. 2017 Jan 11;9(1):55. doi: 10.3390/nu9010055 (PMC5295099; doi:10.3390/nu9010055)
Supplement: Supplementary file 1 [file nutrients-09-00055-s001.docx]

Supplementary Materials: A High Rate of
Non-Compliance Confounds the Study of Whole Grains and Weight Maintenance in a Randomised Intervention Trial—The Case for Greater Use of Dietary Biomarkers in Nutrition Intervention Studies

Mette Kristensen, Xavier Pelletier, Alastair B. Ross and Frank Thielecke

**Table S1.** Overview of intervention foods including whole-grain content and alkylresorcinol content used in the study in the wholegrain and refined grain groups; WG: whole grain; AR: alkylresorcinol; DM: dry matter.

| **Wholegrain Foods** | | | |
| --- | --- | --- | --- |
| **Name** | **Type of WG** | **Proportion of WG (% of DM)** | **AR Content (µg/g DM)** |
| Wholemeal bread | Wheat | 75 | 300 |
| Bulgur | Wheat | 100 | 312 |
| Whole grain couscous | Wheat | 100 | 402 |
| Brown parboiled rice | Rice | 100 | 0 |
| Brown basmati rice | Rice | 100 | 0 |
| Whole grain spaghetti | Wheat | 100 | 214 |
| Whole grain penne pasta | Wheat | 100 | 193 |
| Whole grain rusks | Wheat | 54 | 340 |
| Whole grain crispbread | Rye | 73 | 704 |
| Whole grain crackers | Wheat | 30 | 193 |
| Breakfast cereal 1 | Wheat | 54 | 369 |
| Porridge | Oat | 100 | 0 |
| Muesli 1 | Oat & wheat | 74 | 199 |
| Muesli 2 | Oat & wheat | 65 | 142 |
| Cereal bar 1 | Oat & wheat | 43 | 50 |
| Cereal bar 2 | Oat & wheat | 38 | 32 |
| Cereal bar 3 | Wheat | 26 | 155 |
| Cereal bar 4 | Oat & wheat | 40 | 59 |
| **Refined Grain Foods** | | | |
| **Name** | **Type of Grain** |  | **AR Content (µg/g DM)** |
| White bread | Wheat |  | 28 |
| Couscous | Wheat |  | 69 |
| White parboiled rice | Rice |  | 0 |
| White basmati rice | Rice |  | 0 |
| Spaghetti | Wheat |  | 50 |
| Penne pasta | Wheat |  | 41 |
| Rusks | Wheat |  | 34 |
| Toast | Wheat |  | 34 |
| Corn flakes | Corn |  | 0 |
| Wheat/rice flakes | Wheat & rice |  | 7 |
| Chocolate-flavoured breakfast cereal | Rice |  | 0 |
| Honey coated breakfast cereal | Corn |  | 0 |


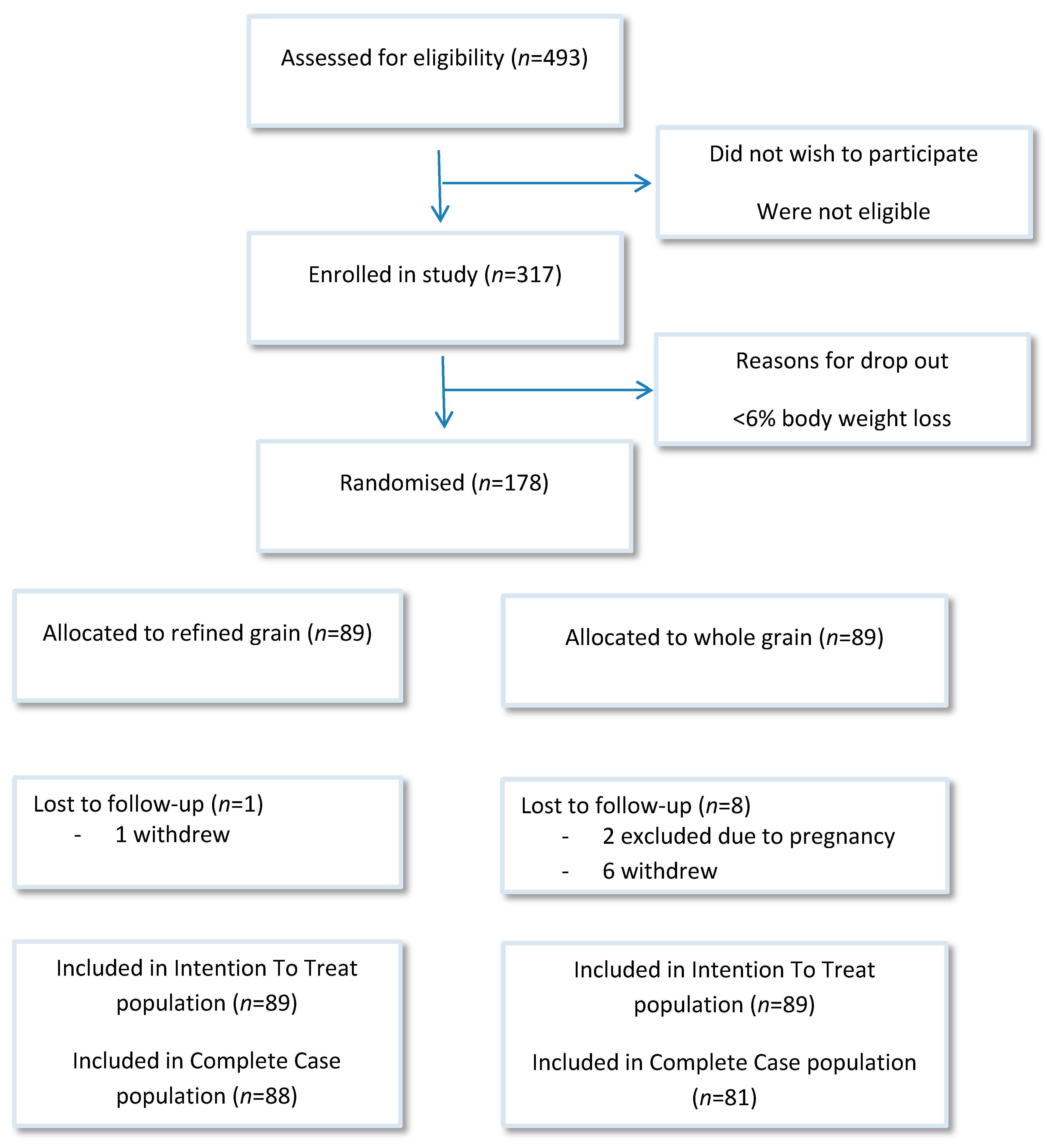


**Figure S1.** Flow of participants through the study.
